# Sclerostin Stimulates Osteocyte Support of Osteoclast Activity by a RANKL-Dependent Pathway

**DOI:** 10.1371/journal.pone.0025900

**Published:** 2011-10-04

**Authors:** Asiri R. Wijenayaka, Masakazu Kogawa, Hui Peng Lim, Lynda F. Bonewald, David M. Findlay, Gerald J. Atkins

**Affiliations:** 1 Bone Cell Biology Group, Discipline of Orthopaedics and Trauma, University of Adelaide, and the Hanson Institute, Adelaide, Australia; 2 University of Missouri - Kansas City School of Dentistry, Department of Oral Biology, Kansas City, Missouri, United States of America; Universidade Federal do Rio de Janeiro, Brazil

## Abstract

Sclerostin is a product of mature osteocytes embedded in mineralised bone and is a negative regulator of bone mass and osteoblast differentiation. While evidence suggests that sclerostin has an anti-anabolic role, the possibility also exists that sclerostin has catabolic activity. To test this we treated human primary pre-osteocyte cultures, cells we have found are exquisitely sensitive to sclerostin, or mouse osteocyte-like MLO-Y4 cells, with recombinant human sclerostin (rhSCL) and measured effects on pro-catabolic gene expression. Sclerostin dose-dependently up-regulated the expression of receptor activator of nuclear factor kappa B (RANKL) mRNA and down-regulated that of osteoprotegerin (OPG) mRNA, causing an increase in the RANKL∶OPG mRNA ratio. To examine the effects of rhSCL on resulting osteoclastic activity, MLO-Y4 cells plated onto a bone-like substrate were primed with rhSCL for 3 days and then either mouse splenocytes or human peripheral blood mononuclear cells (PBMC) were added. This resulted in cultures with elevated osteoclastic resorption (approximately 7-fold) compared to untreated co-cultures. The increased resorption was abolished by co-addition of recombinant OPG. In co-cultures of MLO-Y4 cells with PBMC, SCL also increased the number and size of the TRAP-positive multinucleated cells formed. Importantly, rhSCL had no effect on TRAP-positive cell formation from monocultures of either splenocytes or PBMC. Further, rhSCL did not induce apoptosis of MLO-Y4 cells, as determined by caspase activity assays, demonstrating that the osteoclastic response was not driven by dying osteocytes. Together, these results suggest that sclerostin may have a catabolic action through promotion of osteoclast formation and activity by osteocytes, in a RANKL-dependent manner.

## Introduction

Sclerostin (SCL) is the product of the *SOST* gene, mutations in which cause the high bone mass disease in humans, sclerosteosis [1]. Deletion of *SOST* in mice causes a similar high bone mass phenotype [2]. Partial deletion of a regulatory region approximately 35 kb distal to the *SOST* gene is causative of the high bone mass phenotype seen in Van Buchem's disease [3], [4], [5]. These findings together indicate that SCL has a key role in the regulation of bone mass. SCL has been identified as a target for osteoporosis treatment, with neutralizing antibody and small molecule inhibitor approaches being pursued [6]. The signalling pathways, through which SCL acts, are incompletely understood [7], [8], [9], [10], [11], although it has inhibitory actions on bone morphogenetic protein (BMP) signalling [8], [12] and blocks canonical wingless integration (Wnt) signalling by binding to the Wnt co-receptors, low density lipoprotein receptor (LRP)-5 and 6 [13], [14]. More recently, LRP4 has also been implicated as a major receptor for SCL [15], [16]. We reported recently that SCL stimulated a p42/p44 mitogen activated protein kinase (MAPK) response in human primary osteoblasts, suggesting the existence of additional pathways of sclerostin activity than those described [17].

While attention has been generally focused on the anti-anabolic actions of SCL, existing evidence suggests actions also on bone resorption. Analysis of ovariectomized (OVX) rats treated with neutralizing antibody to SCL showed protection against bone loss and this was associated with a marked decrease in the histomorphometric parameter osteoclast surface, below the level seen in sham OVX animals [18]. A study in 10 month-old intact female rats revealed a bone formation effect of anti-SCL treatment and this was associated with a dramatic inhibition of osteoclastic activity (eroded surface) in these animals [19], consistent with an effect of sclerostin on the osteoclast compartment. Furthermore, a recent report of a phase I clinical trial in healthy human subjects showed that a single subcutaneous or intravenous dose of a neutralizing antibody to sclerostin resulted in a rapid and significant reduction of the serum resorption marker, serum C-telopeptide of collagen (sCTx) [6]. These findings are consistent with the activity of sclerostin as a Wnt inhibitor, since osteoprotegerin (OPG), a potent inhibitor of the pro-osteoclastogenic RANKL-RANK signalling pathway, is expressed in response to canonical [20] and potentially non-canonical [21] Wnt signalling.

Sclerostin is expressed primarily by osteocytes *in vivo* [22], [23]. Parathyroid hormone (PTH) treatment [24], [25] and mechanical loading [26] likely exert an anabolic effect, at least in part, by suppressing SCL expression. In contrast, catabolic stimuli appear to increase SCL expression in bone, for example mechanical unloading [23] or exposure of osteoblast-lineage cells to pro-inflammatory cytokines such as tumour necrosis factor-alpha (TNFα) and TNF-related weak inducer of apoptosis (TWEAK) [17], [27]. Osteocytes can express RANKL *in vivo* [28], [29], [30]. Furthermore the osteocyte cell line, MLO-Y4, can express RANKL and support osteoclastogenesis [31], [32]. We have established a model of human osteocytes, with many of the molecular and phenotypic characteristics of osteocytes, by allowing normal human bone derived osteoblastic cells (NHBC) to differentiate in long-term (4 to 5 weeks) culture [33], [34], [35]. We have also recently identified the osteoblast to osteocyte transition as an important physiological target for SCL [36].

In this study, we examined the effects of SCL on the expression by pre-osteocytes/osteocytes of osteoclastogenesis associated genes. We found that SCL confers a pro-osteoclastogenic phenotype on both human primary pre-osteocytes and the mouse osteocyte-like cell line, MLO-Y4 [37]. We report that SCL stimulates osteocyte support of osteoclastogenesis and does so in a RANKL-dependent manner. We conclude that SCL may have a catabolic action in bone and that this in part explains the dramatic anabolic effect of the natural or clinical neutralization of SCL activity.

## Materials and Methods

### Ethics Statement

The use of all normal human donor derived material was approved by the human ethics committees of the Royal Adelaide Hospital/University of Adelaide and the Red Cross Society of Australia (Approval No.'s RAH090101 & 10-04SA-10). All human material was obtained with informed written donor consent, as required and approved by the respective ethics committees. The use of animals in this study was approved by the animal ethics committees of the Institute of Medical and Veterinary Science and of the University of Adelaide (Approval No.'s 159/08 & ST05/10). Animals were maintained and treated in accordance within the strict ethical guidelines of these committees.

### Recombinant cytokines and antibodies

Recombinant human (rh) sclerostin (rhSCL) and OPG (rhOPG) were purchased from R&D Systems (Minneapolis, MN, USA). Recombinant human RANKL and M-CSF were purchased from Chemicon (Temecula, WA, USA).

### Cells and culture media

Adult human primary osteoblasts (normal human bone-derived cells; NHBC [38]) were isolated from femoral neck trabecular bone and passaged, as we have described previously [39]. MLO-Y4 cells were passaged solely on type I collagen-coated plates, as described previously [37]. Mouse splenocytes were isolated and prepared as described previously [40]. Human peripheral blood mononuclear cells (PBMC) were obtained from normal buffy coats and isolated using Lymphoprep™ (Nycomed Pharma, Oslo, Norway), as described previously [41].

### In situ Immunofluorescence

NHBC were seeded into chamber slides (Lab-Tek, Nunc, Naperville, IL, USA) and cultured for the times and under the conditions indicated. Cells were then rinsed with PBS, fixed with 4% paraformaldehyde in PBS for 10 min on ice, rinsed with PBS and permeabilised with 0.1% Triton X100 for 5 min. Non-specific binding sites were blocked with PBS containing 10% goat serum for 30 min at room temperature. The cells were incubated for 30 min with primary antibodies. Following 3 washes in PBS, cells were incubated with either anti-mouse IgG-FITC or anti-rabbit IgG-FITC for 45 min in a dark humidified container. Cells were then washed in PBS and mounted (Prolong Gold with DAPI anti-fade mounting media; Invitrogen). Samples were examined by confocal microscopy on a Radiance 2100 confocal microscope (Bio-Rad Microscience Ltd, UK).

### Preparation of total RNA and RT-PCR

Total RNA was extracted from NHBC and cell lines treated as above, and complementary DNA (cDNA) was prepared, and gene expression analyzed by real-time RT-PCR as we have described previously [42]. Relative expression between samples was calculated using the comparative cycle threshold (C_T_) method (ΔC_T_), using either 18S rRNA or GAPDH as reference genes, as indicated and as we have published [40], [42]. Oligonucleotide primers were designed in-house to flank intron-exon boundaries, and were purchased from Geneworks (Thebarton, SA, Australia). Real-time oligonucleotide primers for the amplification of human GAPDH, DMP1, SOST RANKL, OPG, 18S and mouse RANKL mRNA species were described previously [34], [42]. Sequences used for real-time RT-PCR amplification of mouse OPG mRNA (122 bp product) were AGCTGGAACCCCAGAGCGAA (sense) and GCAGGAGGCCAAATGTGCTG (antisense).

### Gene expression experiments

To determine the ability of NHBC to form a mineralised matrix, a modification of a method reported previously [43] was used. Cells were cultured in triplicate in wells of a 96 well plate at 8×10^3^ cells/well for NHBC or 5×10^3^ cells/well for cell lines, in αMEM-10 containing dexamethasone (10^−8^ M) and KH_2_PO_4_ (1.8 mM) in the presence or absence of rhSCL. Mature, late osteoblast/osteocyte-like cultures of NHBC were generated by culturing for 35 days in mineralisation medium, as previously described [36]. Cells were then treated with rhSCL (1, 10 or 50 ng/ml) for 3 or 7 days, and RNA prepared as described above. Media containing all supplements were replaced every 4 days and cells were cultured for up to 6 weeks before measurement of cell layer-associated Ca^2+^ levels, as described previously [43].

### Osteoclastogenesis Assays

MLO-Y4 cells were seeded at a density of 2.1×10^4^ cells/cm^2^ into type I collagen-coated wells of a 96-well plate for the purposes of histological staining for tartrate resistant acid phosphatase (TRAP). Alternatively, cells were seeded at an identical density onto slices of whale dentine or into wells of Osteologic™ slides for the purpose of quantification of resorptive activity [40], [41]. Following overnight incubation in seeding medium, media were replaced with those containing varying concentrations of rhSCL. After 3 days of priming under these conditions, splenocytes or PBMC were added (5.3×10^5^ cells/cm^2^) in medium containing the respective priming medium with additional rhM-CSF (25 ng/ml). Control cultures included monocultures of MLO-Y4 cells, splenocytes or PBMC, plated with M-CSF and the same dose range of rhSCL used for co-cultures, and also monocultures of splenocytes and PBMC plated in the additional presence of rhRANKL (100 ng/ml). Media were replaced every 3 days thereafter. Osteoclast number was assessed by TRAP staining after 6–9 days, as described previously [40]. Resorption was assessed 14 days following the addition of osteoclast precursors by von Kossa staining of the mineralised surface and quantification using ImageJ software, as described previously [40].

### Caspase activity assays

MLO-Y4 cells were seeded into collagen-coated wells at an identical density, 2.1×10^4^ cells/cm^2^, to that used for osteoclast-forming co-cultures, as described above. Cells were treated with rhSCL or etoposide (Pharmacia & Upjohn, Kalamazoo, MI, USA) at the indicated doses and duration. Caspase activity was assayed by cleavage of zDEVD-AFC (z-asp-glu-val-asp-7-amino-4-trifluoro-methyl-coumarin), a fluorogenic substrate based on the peptide sequence at the caspase-3 cleavage site of poly (ADP-ribose) polymerase [44], essentially as described previously [45]. Briefly, following treatment, media were removed and non-attached cells and cell debris were pelleted by centrifugation at 400×*g* for 10 min. This pellet and the cells remaining in the respective wells were solubilised in buffer consisting of Tris-HCl (5 mM, pH 7.5), EDTA (5 mM) and NP40 (0.5% v/v), for 15 min at 4°C. Aliquots (20 µl) of cell lysates were added to fresh tubes containing 1 ml of protease buffer (50 mM HEPES, 10% sucrose, 10 mM DTT, 0.1% CHAPS, pH 7.4) and zDEVD-AFC (8 µM, Kamiya Biomedical Company, Seattle, WA, USA). Following incubation for 4 h at room temperature, the resulting fluorescence was measured (LS 50 Spectrofluorimeter, Perkin Elmer, San Jose, CA, USA).

### Assessment of nuclear morphology

MLO-Y4 cells were seeded on plastic chamber slides (Nunc) at the same density as for co-culture experiments, as described above, and stimulated as indicated. After two washes with PBS, cells were fixed in methanol for 5 min, washed again with PBS, and incubated with 0.8 mg/ml of 4′,6-diamidine-2′-phenylindole dihydrochloride (DAPI, Roche Diagnostics, Castle Hill, NSW, Australia) in PBS for 15 min at 37°C. After several washes in PBS, the coverslips were mounted on PBS/glycerin. DAPI staining was visualised by confocal microscopy, as above.

### Statistical Analysis

Student's t-Test was used to analyse differences in mineralisation and cell proliferation experiments. One way analysis of variance (ANOVA) followed by Tukey's post-hoc analysis was used to examine differences in gene expression studies. A value for *p*<0.05 was considered to be significant.

## Results

### Effect of exogenous sclerostin on gene expression during osteoblast differentiation

**Figure 1:**
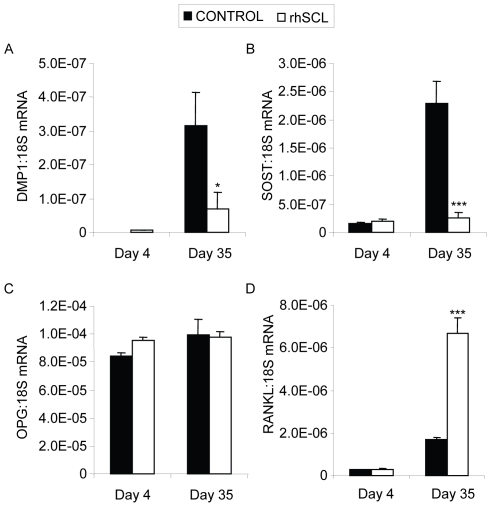
Effects on gene expression of continuous exposure of mineralizing NHBC cultures to rhSCL. NHBC were cultured under mineralizing conditions for up to 35 days in the absence or presence of rhSCL at 50 ng/ml. Media were replenished every 3–4 days. At the time points indicated, total RNA was prepared and real-time RT-PCR performed to determine mRNA expression of A) *DMP1*, B) *SOST*, C) *OPG* and D) *RANKL*. Data shown are means of triplicate reactions ± SD normalized to expression of 18S mRNA. Significant differences to untreated control are indicated by * *p*<0.05 and *** *p*<0.001. Similar results were obtained from 3 independent experiments using NHBC from different donors.

To determine the effect of continuous exposure to exogenous SCL on gene expression in human osteoblasts, we performed real-time RT-PCR analysis of NHBC treated throughout differentiation, for a period of up to 35 days, under conditions otherwise permissive for mineralisation. Consistent with our previous studies [33], [35], [36], this analysis showed that osteoblasts acquired the expected gene profile consistent with differentiation into mature cells with an osteocyte-like phenotype, including time-dependent increases in mRNA levels of the markers DMP1 and SOST (Fig. 1A and B), as well as osteocalcin, BSP-1 and PHEX (data not shown). Continuous treatment with rhSCL (50 ng/ml) affected gene expression primarily at the time of mineralisation, consistent with late stage osteoblasts/early osteocytes being targets for SCL action, and in agreement with our previous report [36]. In these cultures, rhSCL strongly inhibited late expression of DMP1 and SOST. rhSCL had little effect on OPG mRNA expression in continuously treated cultures (Fig. 1C). However, rhSCL strikingly up-regulated RANKL mRNA expression in long-term cultures (Fig. 1D).

**Figure 2:**
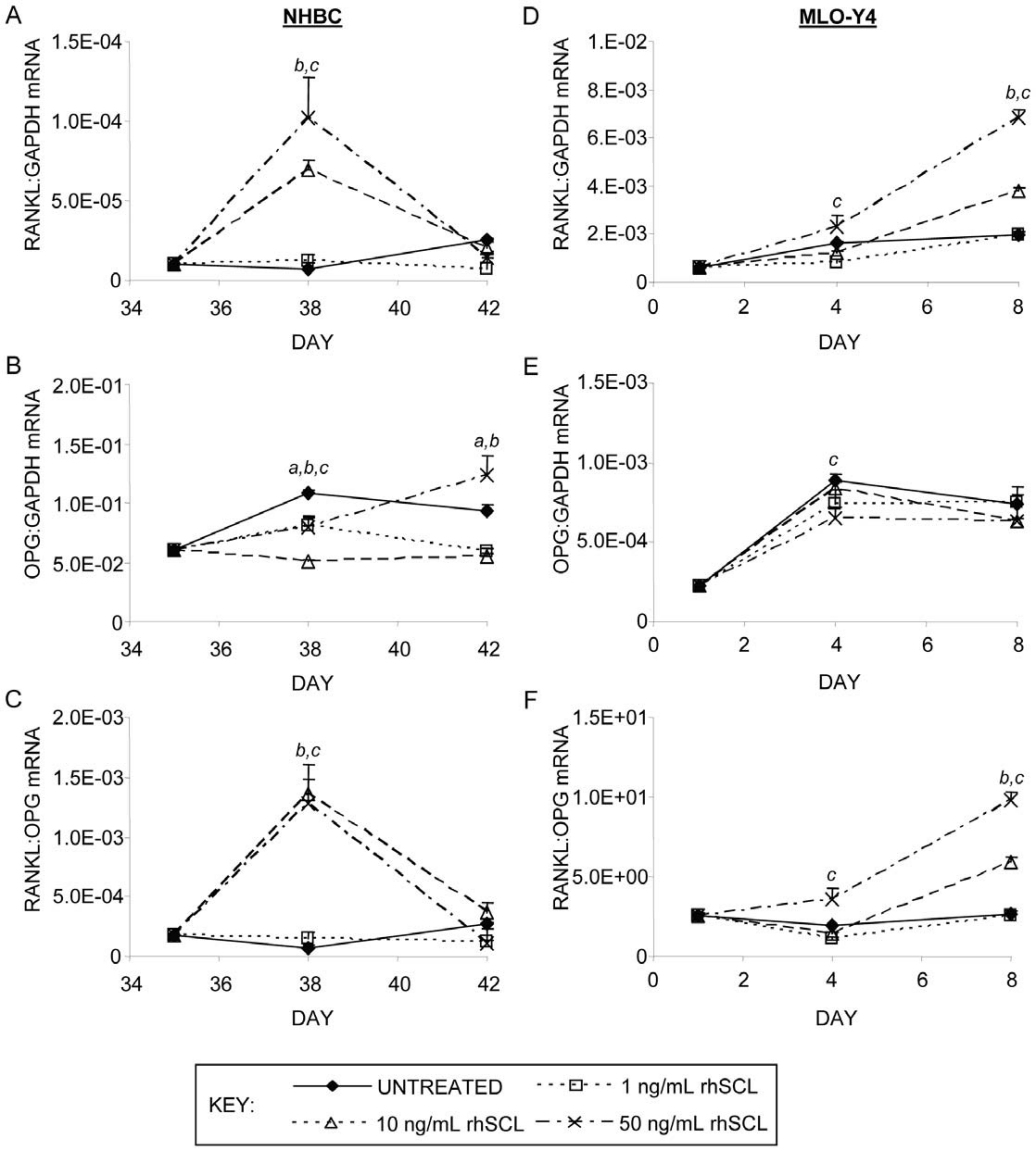
Effects on gene expression of acute exposure of osteocyte-like cells to rhSCL. Human osteocyte-like cultures, derived from NHBC cultured under mineralizing conditions for 35 days, or cultures of MLO-Y4 cells were cultured for 3 or 7 days in the absence of presence of rhSCL at 1, 10 or 50 ng/ml. Media and supplements were replenished at day 3. Total RNA was prepared and real-time RT-PCR performed to determine mRNA expression of A) human *RANKL*, B) human *OPG* or mouse *Rankl* (D) and *Opg* (E). The ratios of RANKL∶OPG mRNA for NHBC (C) and MLO-Y4 (D) were also determined. Data shown are means of triplicate reactions ± SD normalized to expression of *GAPDH* mRNA. *a*, *b* and *c* indicate significant differences (*p*<0.05) to untreated control for rhSCL at 1, 10 and 50 ng/ml, respectively. Near identical results were obtained from 3 independent experiments using NHBC from different donors or MLO-Y4 cells.

Because late-stage cultures exhibited greater sensitivity to sclerostin in terms of osteoclastogenesis-associated gene expression and because we have identified late osteoblasts/pre-osteocytes as SCL targets [36], we next cultured cells under differentiating conditions for 35 days and then treated these acutely with rhSCL. rhSCL dose-dependently increased RANKL mRNA expression after 3 days (mean fold-change with rhSCL at 50 ng/ml of 9.7±4.7, *n* = 3) with levels returning thereafter to those of control cultures in all cases, while the lowest dose of rhSCL used (1 ng/ml) had no effect (Fig. 2A). SCL treatment of these late stage cultures caused a small change in OPG mRNA expression, in the inverse direction to that of RANKL mRNA (mean fold-change with rhSCL at 50 ng/ml of −1.3±0.2, *n* = 3). The decrease in OPG mRNA at 3 days and increase at 7 days of treatment, resulted in a striking transient increase in the RANKL∶OPG mRNA ratio in primary cultures (Fig. 2B and 2C). SCL at 50 ng/ml also increased RANKL mRNA expression in osteocyte-like MLO-Y4 cells, from an already relatively high basal level (Fig. 2D). OPG mRNA levels were decreased in MLO-Y4 cells, resulting in a sustained increase in the RANKL∶OPG mRNA ratio in these cells (Fig. 2E and 2F). Together, these findings implied that SCL can promote a pro-osteoclastic phenotype in osteocyte-like cells.

### Effect of sclerostin on MLO-Y4 support of osteoclastogenesis

**Figure 3:**
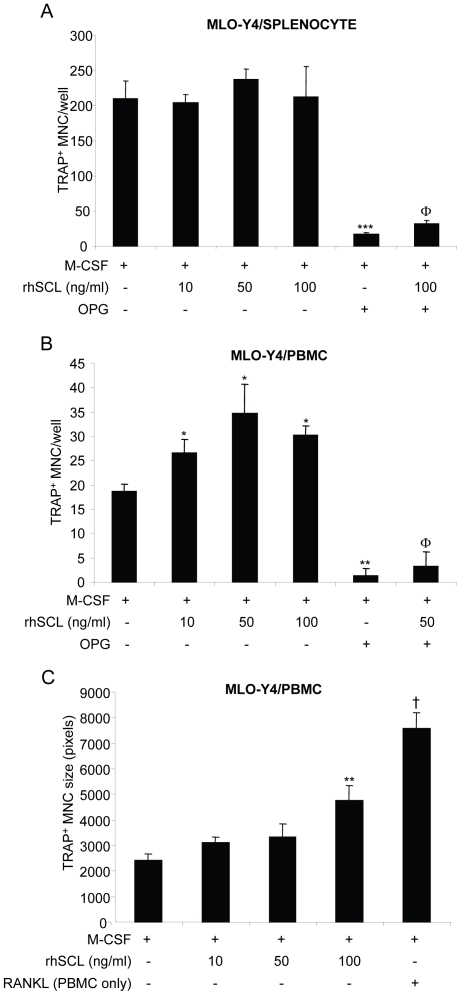
Effects of rhSCL on TRAP^+^ multinucleated cell formation. The effect of rhSCL on osteoclastogenesis was tested in co-cultures of MLO-Y4 cells with A) mouse splenocytes and B) human PBMC. In both cases MLO-Y4 cells were seeded into type I collagen coated culture wells and cultured for 72 h in the absence or presence of rhSCL, as indicated, prior to the addition of either splenocytes or PBMC. All cultures received rhM-CSF at 25 ng/ml. RhOPG was added to some cultures at 100 ng/ml and rhRANKL (100 ng/ml) added to monocultures of either splenocytes or PBMC to confirm the osteoclast-forming potential of these populations (not shown). Media were replenished every 3 days. Cultures were fixed and stained for TRAP at day 6. TRAP-positive MNC, defined as cells with >3 nuclei, were counted from quadruplicate wells. Asterisks denote significant differences to the no SCL control (** *p*<0.01, *** *p*<0.001) and the effect of OPG relative to the corresponding SCL-only treatment is indicated by Φ (*p*<0.001). C) In the case of MLO-Y4/PBMC co-cultures and cells formed in PBMC monocultures treated with rhRANKL, the relative size (in pixels) of TRAP^+^ MNC formed (in each case >50 cells) was measured by Image J analysis. Difference to the no SCL control is indicated by ** *p*<0.01; † indicates difference of rhRANKL control to rhSCL at 100 ng/ml (*p*<0.01). Data shown are representative of at least 3 independent experiments.

Because MLO-Y4 cells express RANKL mRNA at higher levels than NHBC in general, and because they represent a more homogenous cell population, these cells were chosen to test the possibility that SCL treatment could influence their support of osteoclastogenesis. To do so, MLO-Y4 cells were first primed for 72 hours with rhSCL, using a similar dose range that affected RANKL and OPG mRNA expression. Mouse splenocytes or human PBMC were then added to the MLO-Y4 cells and the co-cultures were continued for a further 5–7 days in the additional presence of M-CSF. After this time, cultures were fixed and stained for TRAP activity. Abundant TRAP^+^ cells formed in co-cultures of MLO-Y4 cells with splenocytes, consistent with the relatively high basal expression of RANKL by MLO-Y4 cells, and rhSCL had little effect on the number of TRAP^+^ cells in these cultures (Fig. 3A). SCL moderately increased TRAP^+^ cell number in co-cultures with PBMC (Fig. 3B). In both cases, TRAP^+^ cell formation was strongly inhibited with the addition of OPG, in the presence or absence of rhSCL, showing that osteoclast formation remained RANKL-dependent (Fig. 3). The size of the TRAP^+^ multinucleate cells formed however was larger in the added presence of rhSCL at the highest dose tested (100 ng/ml, Fig. 3C). SCL had no effect on osteoclastogenesis in monocultures of either splenocytes or PBMC treated with M-CSF (not shown).

### Effect of sclerostin on MLO-Y4 support of osteoclast activity

**Figure 4:**
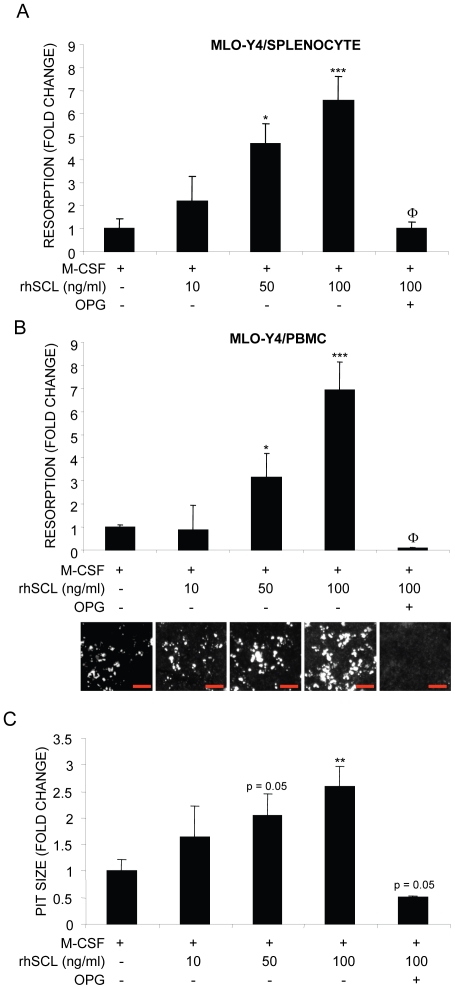
Effects of rhSCL on osteoclast resorptive activity. Co-cultures of MLO-Y4 cells and A) mouse splenocytes and B) human PBMC were established, in which MLO-Y4 cells were seeded onto bone-like Osteologic™ slides and cultured for 72 h in the absence or presence of rhSCL as indicated prior to the addition of either splenocytes or PBMC. All cultures received rhM-CSF at 25 ng/ml and some cultures were treated with rhOPG (100 ng/ml). Media were replenished every 3 days for 14 days. Cells were removed and slides developed as described in Materials and Methods and resorption. The entire surface areas of the developed slides were imaged using an Olympus SZX10 dissecting microscope at high resolution, depicted for the PBMC co-cultures below the corresponding histograms in panel B) (red bars indicate 500 µm). Total resorbed area was then quantified from quadruplicate wells using ImageJ software. C) In the case of MLO-Y4/PBMC co-cultures, mean resorption pit size was determined and expressed as fold-change from the corresponding untreated co-culture. Differences to the ‘no SCL’ control in each panel are indicated by * *p*<0.05, ** *p*<0.01, *** *p*<0.001. The effect of OPG relative to the corresponding SCL-only treatment is indicated by Φ (*p*<0.001). Data shown are representative of 3 independent experiments.

To test the effect of SCL on osteoclast resorption, MLO-Y4 cells were plated onto a mineralised collagen substrate and then treated with rhSCL for 72 hours prior to the addition of either splenocytes or PBMC. Cultures were continued in the additional presence of M-CSF. rhSCL dose-dependently increased the extent of osteoclastic resorption observed after 14 days (significant at 50 ng/ml), in both cases to a maximum of approximately 7-fold at 100 ng/ml rhSCL (Fig. 4A–B). This was accompanied by an increase in mean resorption pit size of 2–2.6-fold, consistent with increased activity of the individual osteoclasts formed in the presence of rhSCL at 50 and 100 ng/ml (Fig. 4C). The increase in osteoclast activity in response to rhSCL was completely inhibited by the co-addition of OPG, indicating that SCL was acting through a RANKL-dependent pathway. In some experiments, conditioned media were taken from MLO-Y4 cells treated with or without rhSCL and tested for their ability to promote osteoclastogenesis; no effect was observed (data not shown), consistent with the requirement for cell-cell contact for the rhSCL effect in this model.[32] Importantly, no direct effects of rhSCL were observed with respect to either TRAP^+^ cell formation or resorption in monocultures of MLO-Y4 cells, splenocytes or PBMC cultured only in the presence of M-CSF (data not shown).

### Effect of rhSCL on MLO-Y4 apoptosis

**Figure 5:**
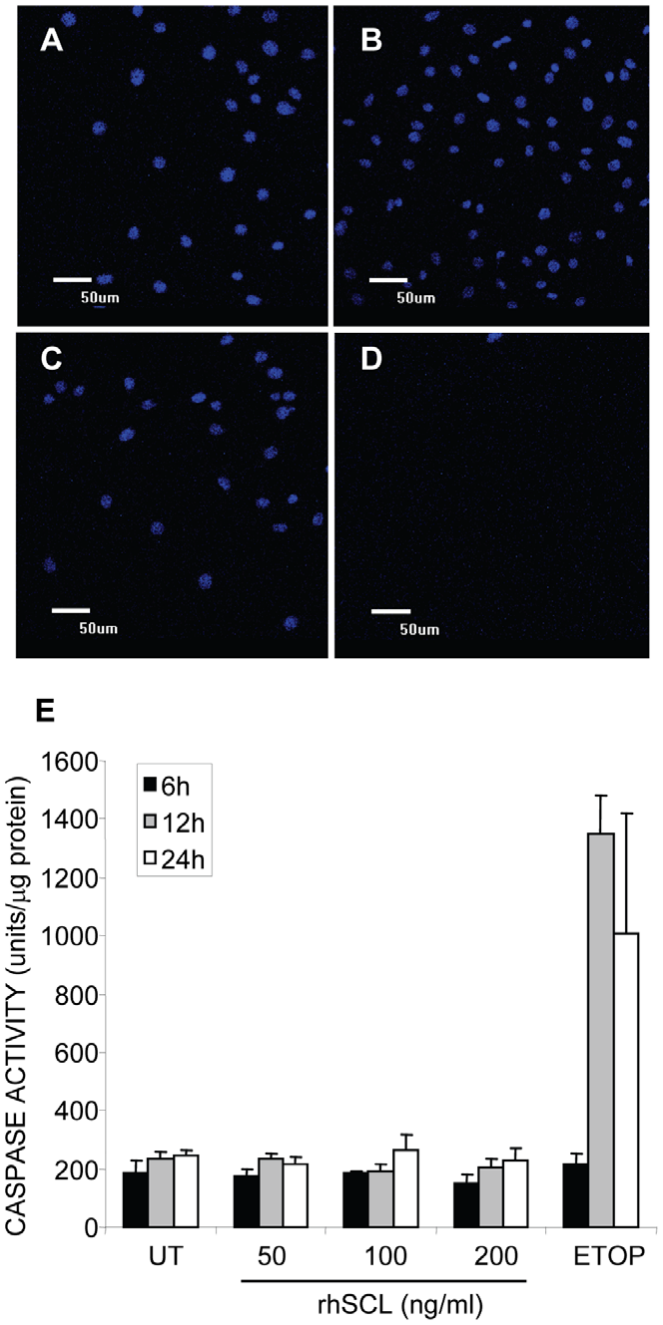
Effect of rhSCL on MLO-Y4 viability. A) Cells were seeded into chamber slides and treated for 3 days A) untreated, or in the presence of rhSCL at 50 ng/ml (B) or 100 ng/ml (C), or D) in the presence of etoposide (50 µM). Cells were then fixed and their nuclear morphology determined by staining with DAPI and visualization by confocal microscopy. E) Cells were seeded into wells of a 6-well plate and cultured for the times indicated with rhSCL (0–200 ng/ml) or in the presence of etoposide (ETOP) at 50 µM. After the times indicated, cell lysates were prepared and caspase 3 activity determined, as described in Materials and Methods. Data are expressed as means of quadruplicate wells ± standard deviations.

Since osteocyte apoptosis has been shown to be a trigger for recruitment and support by osteocytes of osteoclastogenesis, we next investigated the effect of rhSCL on MLO-Y4 cell viability. The effect of rhSCL in this regard was tested against the cytotoxic drug, etoposide at a concentration (50 µM) we shown previously to induce apoptosis in osteoblastic cell lines [45]. Treatment of MLO-Y4 with doses of rhSCL up to100 ng/ml for up to 72 h had no discernible effect on MLO-Y4 viability, as determined by their nuclear morphology (Fig. 5A–D) and by caspase-3 activation assays (Fig. 5E). This implies that SCL is capable of stimulating osteoclastogenesis and osteoclast activity independently of osteocyte apoptosis.

## Discussion

Accumulating evidence suggests that SCL is a key determinant of bone mass in humans and blockage of SCL activity is currently being explored as a novel treatment option for osteoporosis [6], [46], [47]. Because of the high bone mass phenotype of individuals carrying *SOST* mutations, as in the case of sclerosteosis patients, or who lack *SOST* expression, in the case of Van Buchem's disease, it has been assumed that SCL is an inhibitor of bone formation. However, it is possible that SCL also promotes bone catabolism, an activity that is difficult to distinguish definitively from an anti-anabolic activity in the current *in vivo* gene knockout and protein neutralization models. In the current study, we observed that the addition of exogenous rhSCL increased the level of expression of RANKL mRNA and decreased that of OPG, in both cultures of human primary osteoblastic cells differentiated to an osteocyte-like stage and in MLO-Y4 cells. Co-cultures of MLO-Y4 cells primed with rhSCL and either mouse splenocytes or human PBMC, showed that rhSCL promoted osteoclastogenesis. This was associated with moderate increases in the number and size of resulting TRAP^+^ multinucleated osteoclast-like cells. Importantly, the osteoclasts formed in the presence of SCL had greatly increased resorptive activity.

Our findings are consistent with a number of published observations that indicate SCL may have catabolic actions in bone. Li and co-workers demonstrated that mice lacking the *Sost* gene display an osteopetrotic phenotype [48]. In a rat model, neutralization of SCL *in vivo* resulted in protection from OVX-induced bone loss associated with reduced osteoclast surface [18]. More recently, administration of a humanized neutralizing anti-SCL antibody, AMG 785, to a healthy human cohort, rapidly and dose-dependently decreased levels of the bone resorption marker, serum CTx [6].

Consistent with our previous report, SCL in this study had marked effects on pro-osteoclastogenic gene expression in cultures of human primary osteoblasts that were first differentiated into an osteocyte-like phenotype, compared with relatively minor effects on immature cells [36]. The similarity of the response to that observed in MLO-Y4 cells is also consistent with this effect of SCL being on osteocytes. We previously showed that human osteocyte-like cells were sensitive to the anti-anabolic effects of SCL at concentrations as low as 1 ng/ml, concentrations that are higher than but near to the levels reported in human serum of between 0.3 and 0.6 ng/ml [49], [50], [51]. This suggested that cells at the pre-osteocyte and osteocyte stages are major physiological targets for SCL [36]. In the present study, we found that SCL levels of 10 ng/ml or higher produced catabolic activity in these cells, increasing RANKL and decreasing OPG expression. The negative effect of SCL on OPG mRNA expression is consistent with previous reports that SCL is an inhibitor of Wnt signalling and that OPG is expressed in response to Wnt signalling [20], [21]. The regulation of OPG by Wnt signalling may be particularly pertinent to our study as Wnt signalling in osteocytes has been shown to be critical for the control of osteoclastogenesis *in vivo*: Kramer and colleagues [52] demonstrated that conditional deletion of the gene encoding β-catenin in osteocytes, under control of the osteocyte-specific *Dmp1* promoter, resulted in a severe low bone mass phenotype characterised by elevated osteoclastic bone resorption, which was thought to be driven by the observed reduced gene expression of OPG. However, a pathway whereby SCL could directly induce RANKL mRNA expression to a greater extent than was seen on OPG expression mRNA, which appears to be the case in our experimental systems, has not to our knowledge been reported previously. Of note, histomorphometric analysis of *Sost*-overexpressing (transgenic) mice revealed an osteopenic phenotype but with no apparent effect on TRAP^+^ osteoclast number [53], [54]. This latter observation is consistent with our findings using mouse splenocytes that SCL did not increase the numbers of TRAP^+^ cells formed in co-cultures with MLO-Y4 cells, but specifically increased the resorbing activity of the resulting cells. However, serum or urinary analysis of osteoclast activity in *Sost* transgenic animals has not, to our knowledge, been reported. It is also possible that transgenic chronic overexpression of *Sost* triggers a negative feedback effect, which counters the effect on osteoclast activity. This is supported by our observations in human primary osteocyte-like cells that SCL only transiently up-regulated the RANKL∶OPG mRNA ratio cells, and by our published observation that SCL down-regulates the expression at least one of the known receptors for SCL, LRP4 [36]. It will be of interest in future studies to examine acute versus chronic administration of sclerostin *in vivo*. It also remains to be seen which of the proposed receptors for SCL, including the LRPs 4, 5 and 6, are important for the responses observed in our study and whether the effect is dependent on canonical or non-canonical Wnt signalling, or indeed on BMP [8], [12] and/or MAPK [17] signalling.

Overall, our results indicate that in addition to an anti-anabolic effect at lower doses, SCL at high doses may promote a catabolic response by promoting osteoclast activity. Questions that arise from our observations include, how do SCL-responding osteocytes relate spatially to SCL-expressing cells, and, how in turn could this affect osteoclastic bone resorption? In part answer to the latter question, two very recent studies have established the critical importance of osteocyte-expressed RANKL for bone remodelling in the post-developmental skeleton *in vivo*, by targeted deletion of RANKL expression in osteocytes, under control of the *Dmp1* promoter; the loss of osteocyte-expressed RANKL results in a severe osteopetrotic phenotype in these animals [55], [56]. Since the expression of SCL *in vivo* is by mature, mineral embedded osteocytes [8], [22], [57], [58], we propose that neighboring osteocytes, those in closer proximity to endosteal surfaces than osteocytes expressing high levels of SCL [22], may respond to a local, catabolic stimulus-driven increase in SCL. The phenomenon we observed in rhSCL-treated MLO-Y4/PBMC co-cultures of increased activity per osteoclast is consistent with a pro-resorptive response to SCL *in vivo* being spatially restricted to SCL-sensitive osteocytes able to influence osteoclast precursors or osteoclasts already in the process of resorbing bone. Our data suggest that this influence would be at least partly *via* an increased local ratio of RANKL∶OPG in osteocytes.

Osteocyte cell death under certain circumstances is known to be associated with the initiation of bone resorption [59], [60]. Apoptotic osteocytes have been shown to directly stimulate osteoclastogenesis and bone resorption [61]. Prevention of bone fatigue-induced osteocyte apoptosis has been show to block associated osteoclastic resorption [62]. However, glucocorticoid-induced osteocyte apoptosis is associated with reduced osteoclastogenesis [63]. In the current study, we observed no effect of sclerostin at the concentrations used to stimulate osteoclastic activity on MLO-Y4 cell viability over a 3 day treatment period, as assessed by caspase-3 activation assays and examination of nuclear morphology of treated cells. A previous study by Sutherland and colleagues [64] reported that sclerostin could induce apoptosis of human mesenchymal stem cells, but this was at concentrations of between 2–20 µg/ml, 20–200-fold higher than was used in this study and at concentrations we would now consider as supra-physiological. In addition, Kogianni and colleagues [61] reported that osteoclast formation in response to osteocyte apoptotic bodies could not be inhibited with exogenous OPG, whereas in our study the pro-osteoclastic effects of SCL were strictly RANKL-dependent. Thus our results strongly suggest that SCL promotes osteoclast activation by viable osteocytes.

In summary, we have shown that SCL is capable of promoting osteoclastogenesis and osteoclast resorptive activity by an effect on osteocytes, in addition to its anti-anabolic effects. Our findings help to explain the effects on the osteoclast/bone-resorption compartment observed when SCL is neutralized *in vivo*.
